# Development of Mass Spectrometry Selected Reaction Monitoring Method for Quantitation and Pharmacokinetic Study of Stepharine in Rabbit Plasma

**DOI:** 10.1155/2014/961850

**Published:** 2014-02-20

**Authors:** Arthur T. Kopylov, Ksenia G. Kuznetsova, Olga M. Mikhailova, Andrey G. Moshkin, Vladimir V. Turkin, Andrei A. Alimov

**Affiliations:** ^1^Institute of Biomedical Chemistry, 10 Pogodinskaya Street, Moscow 119121, Russia; ^2^Institute of Applied Biochemistry JSC “Biochimmash,” 4 KlaraTsetkin Street, Moscow 127299, Russia

## Abstract

Highly sensitive liquid chromatography mass spectrometry method on triple quadrupole (QQQ) mass spectrometer was successfully applied for pharmacokinetic study of stepharine in rabbit plasma. Specific ion transitions of stepharine protonated precursor ion were selected and recorded in the certain retention time employing dynamic selected reaction monitoring mode. The developed method facilitated quantitative measurements of stepharine in plasma samples in linear range of five orders of magnitude with high accuracy and low standard deviation coefficient and pharmacokinetics parameters were calculated. The apparent volume of stepharine distribution (estimated as ratio of clearance to elimination rate constant, data not shown) allows us to assume that stepharine was extensively distributed throughout the body.

## 1. Introduction

**Figure 1:**
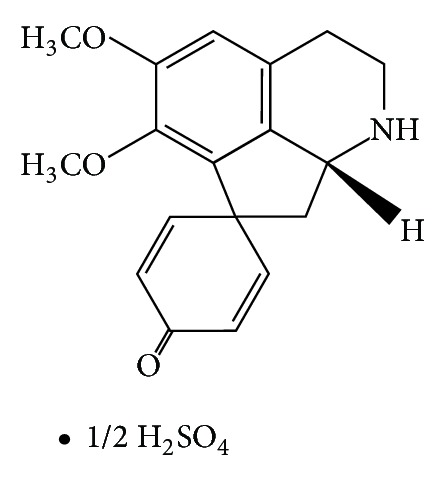
Structural chemical formula of stephaglabrine sulfate (image is adopted from PubChem Compound source).

Stephaglabrine sulfate is a sulfate of isoquinoline proaporphinealkaloid extracted from tuber roots of *Stephania glabra (Roxb.) *of the family Menispermaceae. Stephaglabrinesulfate has the chemical formula (C_18_H_19_NO_3_)_2_·1/2H_2_SO_4_ (Figure 1) and is a white crystal powder with the melting point in the range of 243-244°C, with the subsequent decomposition, soluble in water and aqueous alcohol [1, 2]. Since stephaglabrine sulfate is the dimeric salt, it dissociates to stepharine base with molecular weight 297.1359, which is possible to detect as protonated ion [3].

It has been shown that intramuscular injection of stephaglabrine sulfate in dose of 0.1 mg/kg to rabbits significantly decreases intensity of trophic disturbances of denervated extremities at traumatic injuries of sciatic nerve. Stephaglabrine assists histological neogenesis and regeneration, electrophysiological and functional recovery of nerves after injury [4, 5]. However pharmacokinetic study of the stephaglabrine sulfate still remains poorly investigated.

The main aim of this study is to establish a valid method for detection of stepharine in rabbit plasma after intramuscular administration of the drug. For this purpose, quantitative assay of stepharine has been developed using selected reaction monitoring (SRM). The procedure consists of extraction of stepharine from rabbit plasma, detection by liquid chromatography/tandem mass spectrometry (LC-MS/MS), and measurements of the concentration of stepharine in plasma samples eliciting on the detection of characteristic fragment ions at certain retention time.

Based on the obtained data pharmacokinetic parameters of stepharine were calculated.

## 2. Material and Methods

### 2.1. Materials

Acetonitrile HPLC grade was purchased from Acros (USA), glacial acetic acid was purchased from Merck (Germany), ammonium formate chromatography grade was purchased from Fluka (Germany), ammonia hydroxide was purchased from Acros (USA), chloroform was purchased from Merck (Germany), and deionized water 18.2 mΩ∗cm^2^ was obtained from MilliQ Elix 3 system.

For routine assay calibration, plasma pooled from three rabbits was used. The samples were aliquoted and stored at −80°C. For the matrix effect experiments, plasma pooled from five rabbits was obtained.

### 2.2. Compound

Stephaglabrine sulfate (powder) as analytical compound was obtained from “BioGene Technologies” (Russian Federation).

### 2.3. Animals and Administration of Stephaglabrine Sulfate

All experimental procedures with animals were carried out according the Animal Experimentation Ethics Committee and Veterinary Committee of the RSAU-MTAA (Russian State Agrarian University, Moscow, Russia) guidelines. The Approval Certificate verifying animal experimentation issued by the Animal Experimentation Ethics Committee is number E038-6.176RSAU, issued from November 29, 2012; the Veterinary certificate verifying animal keeping issued by Veterinary Committee is number V812-6.255RSAU, issued from November 26, 2012. The study was acquired on outbred, nonpregnant rabbits aged one year (weight range: 4-5 kg). All animals were kept in a standard animal holding room at Animal Center of RSAU-MTAA. Before the experimental procedure the selected animals were under veterinary supervision for seven days. All rabbits were kept at a standard animal holding room at a temperature of 20 ± 2°C, a relative humidity 65 ± 10%, daylight duration 14–17 hours, and lighting 50–70 luxes. Water and food were *ad libitum*.

The selected rabbits were administrated intramuscular with stephaglabrine sulfate at doses 0.1 mg/kg as it was established before [4]. Each animal received a single dose.

### 2.4. Analytical Instruments and Conditions

Stephaglabrine is the dimeric salt; therefore detection of stepharine compound only (dissociated salt) with *m*/*z* = 298.4 is available LS-MS approach [3]. LC-MS and liquid chromatography with tandem mass spectrometry (LC-MS/MS) analysis were performed on Agilent 6520 Q-TOF mass spectrometer (Agilent, USA) equipped with 1200 HPLC system (Agilent Technologies) nanoflow liquid chromatography system and 6490 Triple Quad mass spectrometer interfaced with 1200 HPLC system microflow liquid chromatography. Evaluation of the compound purity, identity, and full fragment ions spectra was performed on Q-TOF mass spectrometry coupled with chromatographic separation on Zorbax C-18 SB 80 column (75 *μ*m × 43 mm, 5 *μ*m particle size, 80 A pore size). One *μ*L of stephrine sulfate solution (0.1 *μ*g/mL) loaded at flow rate of 2 *μ*L/min in the mobile phase A (12 mM ammonia formate with pH adjusted to 4.55 with glacial acetic acid) for 3.5 minutes. The elution of stepharine was carried out at flow rate of 0.3 *μ*L/min with the gradient of solvents A and B (acetonitrile) starting and maintained at 5% of mobile phase B for 2 minutes and increasing to 95% of solvent B at 20 minutes. Analytical column was washed with 100% of mobile phase B for 7 minutes followed by column equilibration at starting gradient conditions of solvent A-solvent B system (20 : 1, v/v) for 10 minutes. The 6520 Q-TOF mass spectrometer was operated in electrospray ionization at positive mode equipped with HPLC-Chip ion source interface at temperature 340°C. The drying gas (nitrogen) flow rate was 4 L/min, capillary voltage −1952 V, scan rate 3.225 scan/sec at 4 GHz mode, and scan range 100–700 *m*/*z*.

Quantitative analysis was acquired in SRM (selected reactions monitoring) mode on Agilent 6490 triple quadrupole mass spectrometer (Agilent, USA) operated in the positive mode and equipped with Jet Stream ESI ion source. Stepharine was detected in the scheduled selected reaction monitoring (SRM) with unit resolution at both first (Q1) and third (Q3) quadrupoles. The retention time point of stepharine was determined as 9.8 with isolation width of 1 minute for the scheduled SRM method application. The optimal conditions of the analysis were achieved as follows: capillary voltage was −4500 V, nozzle voltage was set at −1200 V, flow rate of drying gas (N_2_) was 16 L/min, flow rate of sheath gas (N_2_) was 8 L/min, temperature of drying gas was set at 340°C, temperature of sheath gas was set at 290°C, and nebulizer gas was operated at 20 psi. The transitions for protonated stepharine [M+H]^+^ 298.4 → 161.2, 192.1, 238.2 were selected for SRM analysis. The isolation window for the fragment ions was set at 0.27 amu, the optimum fragmentor voltage was adjusted to 310 V and collision energy −19 eV. All the samples including calibration points were analyzed in three replications. 30 *μ*L of the samples was injected and loaded on chromatographic column. Calibration was performed on standard samples solution prepared in 20% acetonitrile with concentration of stepharine from 10^−4^ *μ*g/mL to 100 *μ*g/mL. Chromatographic separation was performed on Thermo Hypersil-Keystone ODS column (Thermo Scientific, USA) 100 × 2.1 mm, 5 *μ*m particle size, at flow rate of 200 *μ*L/min and with the following elution conditions: loading onto the column at maintained 20% of mobile phase B for 3 minutes, increasing the gradient to 50% of B at 5 minutes following increasing the gradient to 100% of mobile phase B at 15 minutes. The column was washed and maintained at 100% mobile phase B from 15 to 22 minutes and the composition was decreased to 20% of solvent B at 23 minute. The column was reequilibrated at starting conditions of solvent A-solvent B system (5 : 1, v/v) for 10 minutes at flow 200 *μ*L/min.

### 2.5. Preparation of the Standard Samples of the Stepharine and Determination of LOD and LOQ

The stock solution of stephaglabrine sulfate (1 mg/mL) was prepared by dissolving weighted reference compound in appropriate volume of water-acetonitrile 20% solution. Calibration standards in range of 100, 10, 1, 0.1, 0.01, 0.001, and 0.0001 *μ*g/mL were prepared by dilution of the working solution (100 *μ*g/mL) in of 20% acetonitrile to determine limit of detection (LOD), linearity, and limit of quantitation (LOQ). Each standard sample was analyzed in 10 replicates using developed scheduled SRM method. The fragment ion of stepharine with *m*/*z* = 161.2 was considered as ion quantifier, while fragment ions with *m*/*z* = 192.1 and *m*/*z* = 238.2 were considered as ion qualifiers. Calibration curve was plotted in linear regression fashion. Peaks with signal-to-noise ratio (SNR) more than 7.0 (calculated according to root-mean-square algorithm) and relative standard deviation (RSD) less than 15% were allowed to fit the calibration curve.

### 2.6. Extraction of Stepharine from Rabbit Plasma Samples

0.5 mL of blood samples from each animal was collected from ear vein in the tubes with EDTA before administration and at 15, 30, 60, 90, 120, 180, 480, 720, and 1440 minutes after drug ingestion. Blood samples were immediately put on ice and centrifuged at 5000 g for 10 minutes at 4°C within one hour after blood collection. The obtained plasma was placed in sterile tube and kept at −20°C until analysis.

Plasma proteins were precipitated with acetonitrile. For this purpose, two volumes of acetonitrile were added to 100 *μ*L of rabbit plasma into 1.5 mL plastic tube and 30% ammonia hydroxide was added to adjust the pH to 10. After vortex-mixing for 15 minutes at ambient temperature one volume of chloroform was added three times consequently for 2 hours and incubated at 30°C under regular stirring at 900 rpm at each occasion. The first fraction of upper organic layer was collected after 2 hours of incubation, the second and the third fractions of chloroform extract were collected after additional 30 and 60 minutes of incubation, respectively. The collected fractions were combined and transferred to a new tube and dried under vacuum at 30°C. The resulting pellet was resuspended in 100 *μ*L of 20% water-acetonitrile solution and centrifuged at 14000 rpm for 15 minutes immediately before use. The obtained solution was used for LC-MS analysis.

### 2.7. Selectivity

Selectivity was investigated on serum obtained from 5 different rabbits, which were not administrated with stephaglabrine sulfate (5 blank samples). Rabbit serum samples were treated as described for staphaglabrine sulfate extraction and analyzed in scheduled SRM mode in 10 replicates for possible interfering compounds. Selectivity was evaluated as confident if response level was less than 20% of summarized response of limit of detection and limit of quantitation.

### 2.8. Assessment of Matrix Effect and Extraction Efficiency

Matrix effect was evaluated as ion suppression or ion enhancement. Matrix effect was investigated in plasma by measuring the peak intensities of stepharine in 5 different samples (100 *μ*L) enriched with 0.03 *μ*g/mL of stepharine by postextraction addition in five technical replicates. Samples of rabbit plasma were treated as described for extraction procedure. The obtained organic solvent fractions were dried and resuspended in 100 *μ*L of 20% acetonitrile-water solution. Stephaglabrine was spiked in matrix extracts to give final concentrations of 30 ng/mL and analyzed using scheduled SRM method. Matrtix effect of 15% was accepted. Peak areas of the compound of interest in matrixes with spiked stephaglabrine were compared with peak areas of stepharine of the same concentrations prepared in 20% acetonitrile. Matrix-influence factor *f* has been calculated as

(1) $$\begin{array}{c} f=\frac{A_{\text{add}}-A_{\text{end}}}{A_{\text{aq}}}\times 100, \end{array}$$

where *A*_add_ is the peak area of the compound added in plasma, *A*_end_ the peak area of the endogenous compound, and *A*_aq_ the peak area of the standard compound in water-acetonitrile solution.

The recovery efficiency was evaluated in five different surrogate rabbit plasma samples. Each rabbit plasma was divided in three equal volumes of 100 *μ*L. To one hundred *μ*L of portion of rabbit plasma were added 3, 50, and 100 ng/mL of stepharine to final concentration. Plasma samples were treated with acetonitrile/chloroform solvent system for deproteinization. The resulting extract was dried and resuspended in 100 *μ*L of 20% acetonitrile solution. The recovered amount of stepharine extracted from plasma was measured in 5 replicates. Recovery was estimated by comparison of peak areas of extracted stepharine and peak areas of stepharine measured in standard solutions with the same concentrations. The recovery was calculated according to the formula:

(2) $$\begin{array}{c} R\left(\%\right)=\frac{C_{m}}{C_{a}}\times 100, \end{array}$$

where *C*_*m*_ is the measured concentration of stepharine after extraction; *C*_*a*_ is the known initial concentration of stephaglabrine added in rabbit plasma.

### 2.9. Sample Stability

Freeze-thaw stability of stephaglabrine sulfate solution was evaluated because samples were stored at −20°C. The stability of stephaglabrine pool solutions (in water-acetonitrile 20%) at concentrations of 10 *μ*g/mL, 50 *μ*g/mL, and 100 *μ*g/mL was evaluated after three cycles of overnight freezing following 3 hours of bench thaw at ambient temperature. The loss of stepharine was evaluated by comparison with freshly prepared standard solutions of the same concentrations. Loss of 10% and less was accepted.

### 2.10. Pharmacokinetics Model Design

Calibration curve and pharmacokinetics parameters such as area under the plasma concentration-time curve (AUC) were estimated using trapeziodal method for the observed data and extrapolated to infinity from the last data point using the elimination constant (*K*_el_); the values reported as the maximum plasma concentration (*C*_max⁡_) and the time corresponding to maximum plasma concentration (*t*_max⁡_) are the actual observed ones; the elimination constant (*K*_el_) was calculated as the negative slope of the logarithmic-linear final portion of the plasma concentration-time curve by using linear regression. Half-time of absorption (*t*_1/2_) was determined using elimination constant (ln⁡(2)/*K*_el_). All the parameters were calculated in Mass Hunter Quantitative Analysis B03.02 (Agilent), Sigma Plot (version 9.0), and Microsoft Excel software

(3) ∫15Tmax⁡C0(1−e−k1t)dt+∫Tmax⁡∞C0e−keltdt =C0[(Tmax⁡−15)+((e−k1Tmax⁡−e−15k1)k1)+e−kelTmax⁡kel].

## 3. Results and Discussion

### 3.1. Method Development

#### 3.1.1. Mass Spectrometry Determination of Stepharine

Stephaglabrine sulfate is well-known alkaloid compound originated from plants substrates and represented as the dimeric salt of stepharine [3]. Chemical structure and properties of this molecule were mostly investigated by spectroscopy and spectrophotometry methods [2, 6]. Only a small portion of researches were accomplished using mass spectrometry analysis [7, 8] and no one reported considerate quantitative analysis of stepharine.

**Figure 2:**
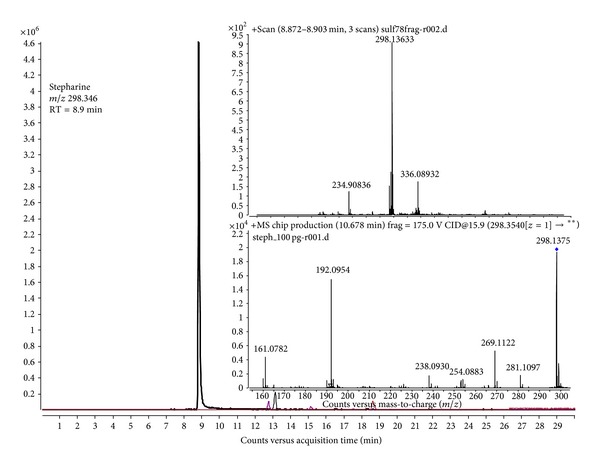
Extracted ion chromatography of stepharine (*m*/*z* = 298.346, retention time 8.9 minutes) precursor registered on high resolution Q-TOF mass spectrometer.

In this study the commercially available stephaglabrine sulfate was analyzed on a high resolution quadrupole time-of-flight mass spectrometer. The obtained data of 100 ng of the compound loaded onto the column demonstrates identification of protonated stepharine precursor ion [M+H]^+^ with high accuracy mass measurements (error 2 ppm) and with *m*/*z* = 298.346 (Figure 2). The content of stepharine in sample makes a total of more than 97.5%, while the traced amount of 2.5% was assigned to auxiliary substances in the regions of 12.7, 15.2, and 18.7 minutes with *m*/*z* = 234.909, 284.183, and 279.153. At the certain chromatographic conditions the retention time point for stapharine was determined as 10.6 minutes.

Fragment ions spectra of stepharine were obtained for confident approval of the certain compound. Solvents adducts corresponding to [M+NH_4_]^+^ or [M+CH_3_COO]^+^ were not observed. Thus, as identity and purity of the obtained commercial compound were determined, we used it further to prepare standard calibration samples and injection form of stepharine for animals. Based on the obtained data of stepharine structure and its fragment ions behavior, the method of quantitative assay on triple quadrupole mass spectrometer was further developed.

#### 3.1.2. Optimization and Validation of Stephaglabrine Extraction

**Figure 3:**
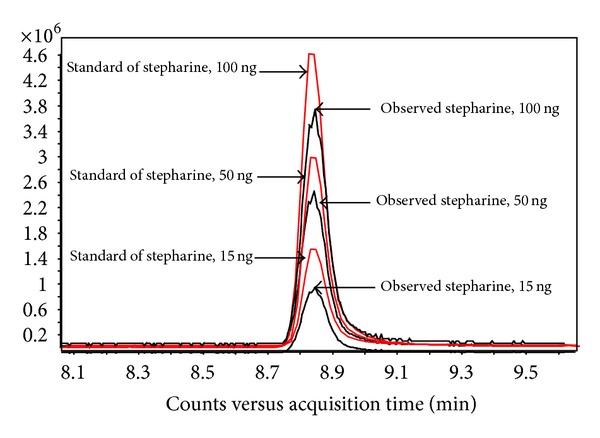
Comparison of peak areas of the spiked standard solutions of stepharine in amounts 15, 50, and 100 ng (solid lines) and observed amount of stepharine after extraction from rabbit plasma (dashed lines). The chromatograms demonstrate peaks of the most abundant fragment ion with *m*/*z* = 161.2.

Efficient extraction of stepharine from rabbit plasma needs to be applied for pharmacokinetic study. There is a little information related to isolation of stepharine from plant tissues and organs [3, 6, 9, 10]. Obviously, these protocols poorly fit the isolation procedure from animal tissues and liquids. In this research we have proposed deproteination of rabbit plasma using acetonitrile/chloroform solvents system highly alkalinized with ammonia hydroxide. To evaluate the recovery of stepharine after extraction, amount of stepharine was added to one hundred *μ*L of rabbit plasma sample to give final concentrations of 3, 50, and 100 ng/mL. Prior assessment of the rabbit plasma was performed to find no interferences with the compound of interest. Stepharine was extracted and the recovery of the extract from rabbit plasma stepharine was evaluated on triple quadrupole mass spectrometer by comparison with peak area of spiked standard stepharine solutions (3, 50, and 100 ng/mL) (Figure 3).

Among several organic solvents which were tested (acetonitrile, methanol, hexane, and dichloromethane), acetonitrile has demonstrated the best recovery yielded at the level of 88.46 ± 4.07% in five replicates (*n* = 5) of each tested sample (Table 1).

As a result, the extracted compound was found as stepharine with *m*/*z* = 298.34 and charge state *z* = 1+. Efficiency of stepharine extraction in methanol solution with pH adjusted to 10 was appreciated at a close level of acetonitrile extraction and yielded 67.5 ± 5.23%. Treatment with dichloromethane and hexane as extracting solvents was depauperated and made mean recovery of 24.4 ± 6.3% (data not shown) which is in consequence of low solubility of stepharine sulfate in highly nonpolar solvents. Thus, based on the successful yield we choose the alkalinized acetonitrile/chloroform solvents system for liquid extraction of stepharine from rabbit plasma. Matrix (rabbit plasma with spiked stepharine) was observed to have no significant ion suppressing effect and consisted of matrix-influence factor *f* in range from −0.07 to −0.09 with maximum RSD = 12.17% in three replicates among all matrix samples. Stability of stepharine demonstrates loss of less than 6% during over three freeze-thaw cycles and mean sample stability was estimated 94.6%.

#### 3.1.3. Development of SRM for Stepharine Quantitative Analysis

**Figure 4:**
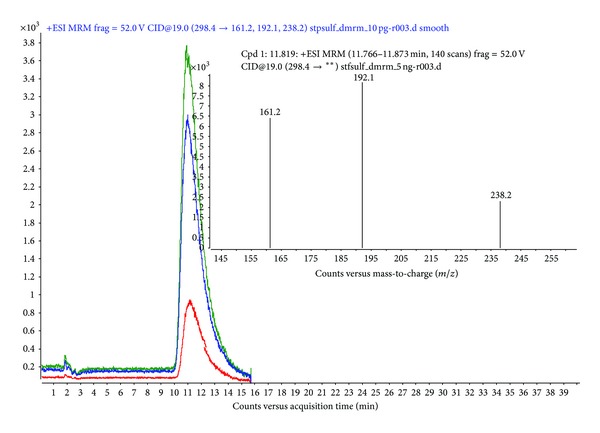
Extracted ion chromatogram (XIC) and SRM-spectrum of 10 pg of stepharine transitions 298.4^1+^ → 161.2^1+^, 192.1^1+^, 238.2^1+^ registered in dynamic selected monitoring mode at the retention time 11.76 minutes.

Since the chromatographic conditions attributed to analysis on triple quadrupole LC-MS system have been changed as described in Section 2.4, the retention time of stepharine is also shifted and defined at 11.6 ± 0.3 minute. Combination of low pH maintained at 4.55 by addition of acetic acid in association with proton-capturing ammonium formate and rapid increasing the gradient to composition of water/acetonitrile with relatively strong hydrophobic properties caused best reproducibility, peak sharpness, and intensity of the stepharine chromatographic peak. Scheduled selected reactions monitoring (SRM) mode was used for stepharine detection on triple quadrupole which is allowed targeted scanning of stepharine compound. The precursor ion of stepharine compound with *m*/*z* = 298.34 and its fragment ions produced after collision-induced dissociation were monitored at the certain retention time (11.5 ± 1 minute) with narrow isolation width (±0.27 amu) in standard samples (solutions of stephaglabrine in acetonitrile) as well as after extraction from rabbit plasma. The collision energy at the level of −19 eV and fragmentor voltage at −310V were adjusted for the transitions (298.34^1+^ → 161.2^1+^, 192.1^1+^, and 238.2^1+^) to attain high sensitivity, reproducibility, and stability of the signal. Three fragment ions (161.2^1+^, 192.1^1+^, 238.2^1+^) obtained from stepharine precursor ion with *m*/*z* = 298.34^1+^ after collision-induced dissociation were recorded on triple quadrupole mass spectrometer for the quantitative analysis and pharmacokinetic characterization. Selection of the defined fragment ions was based on absence of solvent adducts, and SNR was exceeding 7.0 for all the fragment ions and made 16.7, 12.1, and 13.3 for *m*/*z* 161.2, 192.1, and 238.2, respectively (Figure 4).

At the certain conditions, lowest limit of detection was obtained at the level of concentration 10^−4^ *μ*g/mL. However, at the lowest limit of detection the RSD consisted of more than 27%: therefore we used concentration point of 0.001 *μ*g/mL for quantitative analysis. Thus, a range of five orders of magnitude from 10^−3^ *μ*g/mL to 100 *μ*g/mL was considered for calibration curve plotting and the lowest limit of quantitation was attributed to 0.001 *μ*g/mL with RSD of 2.39%.

**Figure 5:**
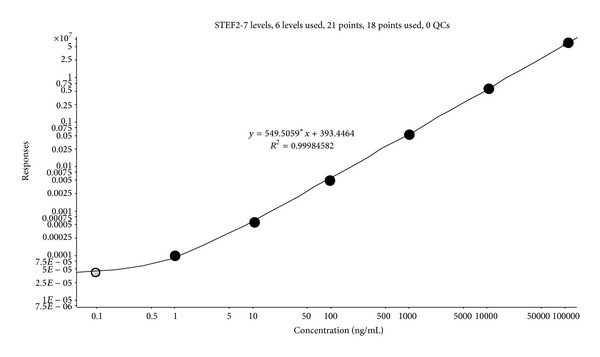
Calibration curve of stepharine plotted in the range of 100 pg/mL to 100 *μ*g/mL. Calibration curve was weighted with 1/*x* factor and the correlation coefficient made *r* ^2^ > 0.99. Each data point averaged on three replicates.

Since no significant intercepts and curvatures were observed within the inspected concentration range, we applied linear model to fit the calibration curve. Heteroscedasticity was evaluated by comparison of covariance of the lowest (0.001 *μ*g/mL) and the highest calibrators (100 *μ*g/mL) and the best fit of linearity was achieved at weighting factor 1/*x*. The achieved linearity of the dependence of peak area of stepharine on concentration in standard samples is demonstrated in Figure 5.

Each calibration point measurement was averaged on five replicates of each standard sample. The Pearson's correlation coefficient within this range was *r* ^2^ = 0.99984. Reproducibility of precursor peak area (RSD 9.3%, replicates *n* = 5, and concentration 0.001 *μ*g/mL) was achieved while injecting stepharine onto the column equilibrated with 20% of mobile phase B (acetonitrile) and followed by a rapid increase of acetonitrile to 50% which caused early elution of nonpolar compounds coextracting with stepharine from crude rabbit plasma extract. Quantitative assessment was accomplished with fragment ion *m*/*z* = 161.2, which was assigned as ion-quantifier.

The concentration of stepharine in rabbit plasma samples after intramuscular administration was determined using calibration curve plotted in linear regression fashion. Content of stepharine extracted from rabbit plasma was analyzed in five replicates of each sample before and after administration.

### 3.2. Pharmacokinetic Parameters of Stepharine Sulfate

Stephaglabrine sulfate affects the synaptic transmission and diminishes frequency of miniature endplate potential at low concentration [10]. It also inhibits cholinesterase and pseudocholinesterase *in vitro* [11] and possesses antihypertensive activity without side effects such as *α*- or *β*-adrenergic blockade, sedative or depressant effect. Pharmacokinetic parameters of stephaglabrine after intramuscular administration were determined. Animals were treated with stephaglabrine sulfate in dose of 0.1 mg/kg and 0.5 mL blood samples were collected subsequently before and after administration of drug as described in experimental section. Measurements of the stepharine in plasma were made immediately after extraction and utilized calibration curve extrapolated in the same testing day. The concentrations of stepharine measured in rabbit plasma at the defined time points are given in Table 2.

**Figure 6:**
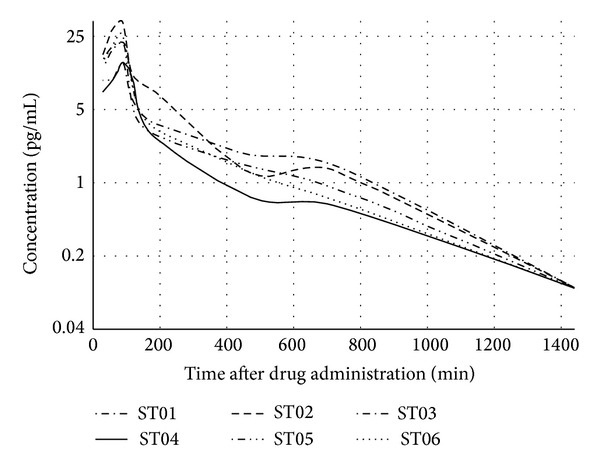
Concentration-time tracking curve of the observed in rabbit plasma stephaglabrine after intramuscular administration.

The effect of stephaglabrine injection on the rabbit plasma is demonstrated on the tracking time-concentration curves (Figure 6). The concentration-time curves show that plasma concentration of alkaloid varied more than 10-fold from the baseline. It should be noted that among all the studied animals stepharine varied in narrow concentration ranged from 5.7 to 8.1 ng/mL in first fifteen minutes after administration. The maximum concentration (*C*_max⁡_) of the drug was observed at 90 minutes in all cases. Registered maximum concentration fluctuated in a few more wide limits: from 13.9 to 33.9 ng/mL that apparently related with the individual particularities of the animals. However, the minimum concentration of the substance was discovered through 12 hours in all analyzed cases.

The observed close values of elimination rate constants (*K*_eq_) suggested the common mechanism of excretion. After 24 hours of intramuscular administration stepharine was not found in plasma samples. Thus, considering stated generality in maximum concentration reached and declination fashion one can assume that stepharine was rapidly cleared from the body. The pharmacokinetic parameters of stephaglabrine sulfate are shown in Table 3.

Determination of the initial concentration (*C*_0_) of stepharine in plasma showed that after intramuscular administration of stephaglabrine sulfate the absolute values may vary among the tested rabbits, which is probably caused by the individual particularities of the animals, in part, due to differences in absorption rate (*K*_1_). The total body clearance (CL) was calculated as the ratio of injected dose to AUC_15→∞_ value and ranged from 118 to 226 mL/min in case complete stepharine absorption is assumed.

The estimated terminal phase half-life (*T*_1/2_) ranged from 99 to 139 minutes. Half-life estimation is sensitive to the number of data points, as well as to the final point of time in the terminal stages. In part, half-life depends on the type of distribution throughout the body. Although, little is known about the metabolic pathways of stepharine, but an affinity of the drug to serum proteins can be assumed [12]. The apparent volume of stepharine distribution (estimated as ratio of clearance to elimination rate constant, data not shown) allows us to assume that stepharine was extensively distributed throughout the body.

## 4. Conclusion

In this research we have developed and validated highly sensitive liquid chromatography/tandem mass spectrometry method for detection and quantitation of stepharine in rabbit plasma after intramuscular administration and designed pharmacokinetic model. The extraction of stepharine from plasma samples was optimized and yielded more than 66% of stepharine. The identity of the parent drug extracted from plasma was confirmed by high resolution Q-TOF (quadrupole time-of-flight) and quantitative analysis was assessed by selected reactions monitoring on QQQ mass spectrometers by three characteristic transitions (298.4 → 161.2, 192.1, 238.2) of stepharine protonated precursor ion. Calibration curve of stepharine peak areas against its concentration was plotted in the range from 0.001 *μ*g/mL to 100 *μ*g/mL with standard deviation ±3%. The obtained pharmacokinetics data suggested that stepharine exhibits extensive distribution and rapidly cleared from the body.

## Figures and Tables

**Table 1:** Stepharine extraction recovery from rabbit plasma using acetonitrile and methanol.

Extracting organic solvent	Concentration of stepharine in preextracted plasma, *μ*g/mL	Postextraction recovery, % ± RSD
Acetonitrile	3	85.4 ± 5.2
	50	87.8 ± 3.3
	100	92.2 ± 3.7

Methanol	3	67.1 ± 4.7
	50	66.3 ± 6.1
	100	69.1 ± 4.9

*The results are averaged on five replicates (*n* = 5).

**Table 2:** Concentration of stepharine in rabbit plasma after intramuscular administration*.

Time after administration, minutes	Animal internal ID					
	ST01	ST02	ST03	ST04	ST05	ST06
	Measured mean concentration of stephaglabrin in plasma, ng/mL					
0	0	0	0	0	0	0
15	8.1 ± 0.6	7.9 ± 0.2	6.8 ± 0.4	5.7 ± 0.2	6.3 ± 0.2	7.0 ± 0.3
30	9.4 ± 0.5	16.5 ± 0.8	14.8 ± 0.2	7.3 ± 0.3	12.7 ± 0.3	13.9 ± 0.2
60	10.0 ± 0.3	29.3 ± 2.2	19.5 ± 0.6	9.5 ± 0.5	18.5 ± 0.2	20.4 ± 0.2
90	13.9 ± 0.9	33.9 ± 2.8	20.4 ± 1.7	14.2 ± 0.2	21.2 ± 0.2	25.8 ± 0.1
120	5.8 ± 0.1	10.4 ± 0.5	6.5 ± 0.1	9.7 ± 0.2	5.1 ± 0.3	7.6 ± 0.09
180	3.8 ± 0.1	7.8 ± 0.3	n/a	2.8 ± 0.2	2.9 ± 0.07	3.3 ± 0.08
480	1.8 ± 0.02	1.2 ± 0.02	n/a	0.7 ± 0.01	1.4 ± 0.08	1.2 ± 0.08
720	1.5 ± 0.02	1.3 ± 0.01	n/a	0.6 ± 0.01	0.9 ± 0.04	0.7 ± 0.02
1440	0	0	n/a	0	0	0

*Measurements were made up by using calibration curve and averaged on five replicates.

**Table 3:** Pharmacokinetic parameters of stephaglabrine in rabbit plasma samples after intramuscular injection.

Animal (ID)	Dosage, mg	*C*_max⁡_, ng/mL	*T*_max⁡_, min	*K*_1_, min^−1^	*K*_el_, min^−1^	*T*_1/2_, min	*C*_0_, ng/mL	AUC_15_, ng∗min/mL
ST01	0.50	13.9 ± 0.9	90	0.01	0.005	139	10.57	1661
ST02	0.50	33.9 ± 2.8	90	0.025	0.006	115	21.4	3187
ST03	0.45	20.4 ± 1.7	90	0.02	n/a	n/a	n/a	n/a
ST04	0.45	14.2 ± 0.2	90	0.013	0.007	99	22.5	2516
ST05	0.50	21.2 ± 0.2	90	0.02	0.006	118	15.3	2217
ST06	0.50	25.8 ± 0.1	90	0.023	0.007	121	17.1	2769
